# The use of phoxim and bendiocarb for control of fleas in farmed mink (*Mustela vison*)

**DOI:** 10.1186/s13028-018-0412-6

**Published:** 2018-09-22

**Authors:** Kim Søholt Larsen, Martin Sciuto, Jan Dahl

**Affiliations:** 1 KSL Innovation ApS, Ramløsevej 25, 3200 Helsinge, Denmark; 2 KSL Consulting ApS, Lejrvej 17, 1, 3500 Værløse, Denmark; 3 Jan Dahl Consult, Østrupvej 89, 4350 Ugerløse, Denmark

**Keywords:** Bendiocarb, Farmed mink, Fleas, Phoxim

## Abstract

**Background:**

Fleas (*Ceratophyllus sciurorum*) are common on farmed mink in Denmark. When present, the fleas have a negative impact on the health of the farmed mink and are of nuisance for farm staff. Severe infestations of fleas cause anemia, poor growth and may result in death of mink kits. Changed behavior of the dams is also observed. Further it has been demonstrated that the fleas are vectors of Aleutian disease virus. Flea control is based on use of a few insecticides and resistance has been reported against permethrin. There is thus a need for new flea control products. In this blinded, randomized clinical trial according to GCP standard, phoxim spray and bendiocarb powder for flea control on mink farms were investigated.

**Results:**

Both the phoxim spray solution and bendiocarb powder were found to be efficient for the control of *C. sciurorum* fleas on farmed mink. Phoxim treatments reduced the number of fleas by 98.4% and the bendiocarb treatments reduced the number of fleas by 99.0% in the mink nest boxes when compared to counts in controls. No clinical signs were observed post treatment.

**Conclusions:**

The study demonstrated that phoxim sprayed on the animals and the use of bendiocarb powder in the nest box material were highly efficient for the control of the *C. sciurorum* fleas on farmed mink. Both products were safe to use at the recommended dose rate. Both compounds are recommended to be integrated in a new farm management plan suggested here.

## Background

Mink farmers are often confronted with flea infestations in mink (*Mustela vison*) and the need of an adequate treatment [1, 2]. The squirrel flea, *Ceratophyllus sciurorum*, is the most common pest on farmed mink in Denmark and several other countries [3]. Severe flea infestations may cause anemia, poor growth and may result in death of the very young mink kits. Furthermore, the flea infested dams may become restless and thus often leave the nest boxes. This is associated with poor care of the kits, starvation and subsequently death. Skin and fur can also be damaged when the mink reacts to the fleas by scratching and biting [4]. A flea problem is typically detected when observing fleas on the mink, finding increased numbers of anemic or dead newborn kits or when the farm personnel is being bitten by fleas. The squirrel flea has also been demonstrated to be the vector of pathogenic organisms, e.g., Aleutian mink disease virus [5]. Control of the squirrel fleas on mink farms is thus of vital importance for the health of the mink.

The squirrel flea is almost only present on the host while blood feeding. After the blood meal they leave their host and are then found in the host’s surrounding, typically in the straw material of the nest box. It is also in the nest box material that the fleas lay their eggs and where the development from egg to adult flea takes place [4].

Only two insecticides are at present registered for flea control in mink farms in Denmark, namely diflubenzuron and permethrin. Both products are talc powder formulated and are spread/dusted in the straw material in the nest box of the mink. These treatments are made preventively but permethrin is also used when fleas are observed on the farms. Failure of controlling the fleas using permethrin has been experienced on farms not only in Denmark [1, 6], but also in other countries [4]. The reduced efficacy observed in Denmark seems to be due to the presence of permethrin resistance [6] but poor management practice on the farms is also part of the flea problem [7]. There is thus a need for finding new well tolerated insecticides for squirrel flea control on farmed mink.

The purpose of this study was to evaluate the efficacy of phoxim and bendiocarb formulations for flea control on farmed mink.

## Methods

### Trial design

Phoxim was applied as a 0.1% phoxim aqueous dilution spray (1.9 mL per 100 cm^2^ body surface) (Sebacil^®^ Vet., Bayer) and bendiocarb as a 1.25% bendiocarb powder (4 g per nest box) (Ficam^®^ D, Bayer) on an established flea infestation. Two control groups (water spray or talc powder without any insecticide, respectively) were included. Each nest box in the treated and the control groups was regarded as a separate unit as the migration of fleas between nest boxes is very limited or does not occur at all. Each cage in the study with one mink was separated from the next by an empty cage. The trial was blinded, by separation of study roles: the dispenser was not involved in any clinical examination throughout the study and the staff counting flea numbers was unaware of the treatment minks had received.

### Farms and animals

The study was designed as a field study and two commercial standard mink farms were selected. Both farms were located in Jutland, Denmark and both farms had a record of squirrel flea problems for several years. The chains of cages were placed under a roof and with a pathway between the chains. Each cage consisted of a wooden nest box (0.075 m^2^) and a wire netting box (0.27 m^2^). All nest boxes were newly packed with barley straw. Below the straw a folded newspaper was placed to avoid material from falling out of the nest box. Food for the mink was applied on the top of the wire netting boxes. The mink used were 160 barren female mink, 1 or 2 years of age. The animals were of different breeds. One unit with 80 mink on each farm was used. The mink were split randomly into two treatment groups. Forty mink were included in the treatment groups (20 mink in each group) and 40 were acting as controls (again 20 mink in each group). The animals were inspected daily by the farm personal during the trial period. Further, a veterinarian also inspected all animals prior to treatment and at the end of the study. Clinical visual inspection of the animal in the wire netting box was performed with special emphasis on the hair and skin prior to the treatment and at the end of the study 7 days later.

### Treatments

The aim of the treatments was either controlling the fleas on the mink or the fleas in the nest box material. The first product tested was 50% w/v phoxim (Sebacil^®^ Vet.) administered as a 0.1% phoxim aqueous dilution sprayed once. Considering the body surface of minks to be similar to those of ferrets (ferrets with a body weight of 0.75 kg and 1.0 kg do have a body surface area of 0.082 m^2^ and 0.099 m^2^, respectively [8, 9]), the topical spray-dosing of the mink were conducted according to Table 1.

**Table 1:** Dosing table for 0.1% phoxim aqueous dilution sprayed on the body surface of mink

Calculation of body surface area (BSA)			Application volume for animals in the body weight range of	
BW in gram	BSA in m^2^	Target volume (mL)	Weight range (g)	IVP (mL)
750	0.082	15.7	750–999	19
1000	0.099	19.1	1000–1249	22
1250	0.115	22.1	1250–1499	25
1500	0.130	25.0	1500–1749	28
1750	0.144	27.7	1750–1999	30
2000	0.158	30.3	2000–2249	33
2250	0.171	32.8	2250–2499	35
2500	0.183	35.1	2500–2749	37

The target volume was calculated based on a recommended dose of 25 mL per adult animal of 1.5 kg body weight. Body surface area (BSA) in m^2^ = K × [body weight (BW) in grams^2/3^] × 10^−4^, K = constant of 9.94 for ferrets

The 0.1% phoxim water-based solution was sprayed on the mink. The second product used was 1.25% w/w bendiocarb powder (Ficam^®^ D), which was applied to the straw material in the nest box at a dose of 4 g. Twenty-five millilitre of water and 4 g of talc powder were used as control references for the phoxim and bendiocarb treatments, respectively. The treatments were performed the day after 50 *C. sciurorum* fleas were placed in each of the nest boxes. The effect of the treatments was measured as the number of fleas found in the material of each nest box 1 week after the treatments.

### Experimental infestation

The barren female mink were transferred to cages with new straw bedding material. No fleas were thus present in the nest boxes before the artificial infestation. All nest boxes were then artificially infested with 50 adult fleas (*C. sciurorum*) on the day before treatment. The fleas used for this study were collected from the same farm where the mink were originating. This was done to prevent any possible transmission of pathogenic organisms between farms. The fleas for each nest box were kept in separate tubes for up to 24 h before the day of use.

### Parasite counting

On study day 7 all material in the nest boxes was transferred into large plastic bags for counting of live fleas within 72 h after collection. Each bag was given a unique code number. The staff performing the flea counting (entomologists) was blinded by means of the coded bags. Counting of fleas was done by looking through the material from each of the nest boxes and counting all adult live fleas found in the material.

### Statistical analyses

Data for both trials were analysed in a loglinear model with treatment group and farm as explanatory variables (PROC GENMOD, SAS Institute, NC, USA). The interaction between farm and treatment was evaluated. Effects are reported per farm, and if the interaction between farm and treatment was non-significant, an overall effect was calculated. Initially a Poisson distribution was assumed, but if the model fit, evaluated as a high Pearson Chi square, indicated a low fit of the model, a negative binomial distribution was assumed. Finally, if overdispersion was still present after this, a post hoc adjustment of P-values and confidence intervals was calculated, using the P-scale option in PROC GENMOD (SAS Institute) (adjusting P-values and confidence-intervals by the Pearson Chi square statistics divided by degrees of freedom).

Results are presented as percentage reduction in each treatment-group, compared to the respective control-group.

Thus, the efficacy of the two compounds included in this study was evaluated on their ability to control fleas present in the mink nesting material compared to untreated controls.

## Results

Three bags with nest material could not be used for flea counting: One sample from one farm had lost the cage code label during the collection process and two samples from the other farm were excluded due to incorrect numbering in the registration process. These three nest boxes were excluded in this study and data from flea counts in 157 nest boxes are thus included in this study.

No animals experienced health problems, no animals received additional medical treatment and no nest material was removed from the nest boxes. No changes in normal behaviour were observed as well as no side effects were observed during the study period.

### Phoxim treatment

Due to overdispersion (scaled Pearson Chi square = 4.73), the Poisson-model was discarded and a negative binomial distribution was assumed. This improved model fit considerably better, but some overdispersion was still present (scaled Pearson Chi square = 1.44). Confidence intervals and P-values were adjusted using the P-scale-option.

Initially an interaction between farm and treatment-effect was examined, giving a non-significant P-value of 0.34. Results are presented as results per farm and in a combined analysis (Table 2).

**Table 2:** Statistics for the phoxim treatment group

Farm number	Number of nests		Nests with fleas		Average number of fleas^a^		Average number of fleas in positive nests^a^	
	1	2	1	2	1	2	1	2
Treatment	20	20	4	5	0.3 (0.1–0.8)	0.4 (0.2–1.0)	1.5 (0.5–4.1)	1.6 (0.7–3.8)
Control	20	20	20	20	24.8 (18.8–32.7)	18.9 (14.2–25.0)	24.8 (18.8–32.7)	18.9 (14.2–25.0)

^a^Assuming a negative binomial distribution

### Bendiocarb treatment

Due to overdispersion (scaled Pearson Chi square = 4.41), the Poisson-model was discarded, and negative binomial distribution was assumed. This improved model fit considerably better, but some overdispersion was still present (scaled Pearson Chi square = 1.41). Confidence intervals and P-values were adjusted using the P-scale-option.

Initially an interaction between farm and treatment-effect was examined, giving a non-significant P-value of 0.08.

Using 0.1% phoxim the reduction in farm 1 was 98.8% and in farm 2, 97.9% with an overall reduction of 98.4% (Tables 3, 4). Use of 4 g of 1.25% bendiocarb in the mink nest boxes, reduced the number of fleas by 98.2% in farm 1 and 99.7% in farm 2. Overall the reduction was 99.0% (Table 5).

**Table 3:** Farm specific flea reductions in phoxim treatment group compared to its control and combined effect

	Farm 1	P-value	Farm 2	P-value	Combined	P-value
Reduction	98.8% (96.7%–99.6%)	< 0.0001	97.9% (94.6%–99.2%)	< 0.0001	98.4% (96.8%–99.2%)	< 0.0001

**Table 4:** Statistics for the bendiocarb treatment group

Farm number	Number of nests		Nests with fleas		Average number of fleas^a^		Average number of fleas in positive nests^a^	
	1	2	1	2	1	2	1	2
Treatment	19	19	2	1	0.3 (0.01–0.7)	0.06 (0.01–0.44)	2.5 (0.8–7.6)	1 (0.1–9.2)
Control	20	19	18	18	14.7 (10.2–20.9)	18.4 (12.8–26.5)	16.3 (12.7–20.8)	19.4 (15.3–24.7)

^a^Assuming a negative binomial distribution

**Table 5:** Farm specific flea reductions in bendiocarb treatment group compared to its control and combined effect

	Farm 1	P-value	Farm 2	P-value	Combined	P-value
Reduction	98.2% (94.0%–99.5%)	< 0.0001	99.7% (98.2%–99.9%)	< 0.0001	99.0% (97.3%–99.7%)	< 0.0001

## Discussion

In the present study the bendiocarb and phoxim containing products were found to control the fleas and to be easy to apply. Phoxim has already been tested for flea control on farmed mink [10]. However, another treatment strategy was used, i.e. using spontaneously infested nest boxes, two treatments of insecticide regime and a dosage not related to the weight of the mink. Due to this, a direct comparison with the present study is not possible.

The number of fleas collected in the untreated control nest boxes after 7 days showed a reduction in the number of fleas of approximately 50% in the control group. A likely explanation would be that some of the fleas infesting the mink are removed by oral grooming. It is known that farmed mink perform oral grooming [11, 12] but the effect of this grooming on the flea population has not been demonstrated. Removal of fleas (*Ctenocephalides felis*) by oral grooming has been observed in, e.g., cats. Here, by grooming, the cats removed between 4.1 and 17.6% of the fleas daily [13]. It was also demonstrated that cats with fleas groomed twice the rate of the flea free cats [14]. Another explanation for the reduced number of fleas could be that the fleas are simply leaving the nest boxes. However, no fleas were observed outside the nest boxes containing the flea hosts when *C. sciurorum* fleas were reared in captivity [15].

This spontaneous reduction of the number of fleas did not affect the validity of the trial as the number of fleas persisting in the control group was sufficient for a meaningful calculation of efficacy. The numbers meet requirements given by the relevant EMEA guideline.

Based on the present results from the phoxim and bendiocarb treatments, these compounds are suitable for flea control. As mink farmers perform management routines on the farm on a yearly basis related to the synchronous life cycle of the mink, a flea management plan could be included in these routines. In a management plan, the mink farmer can treat the fleas efficiently by simple hygiene measures (removing the straw in the nest boxes) in combination with a repeated chemical control (treating the mink and/or the nest boxes with a flea control product, respectively). In this flea management plan the phoxim spraying should be done at specific times of the year when the animals are moved in traps between the cages anyway. The bendiocarb powdering of the nest box material should then be done when the animals are moved to newly packed nest boxes. Both types of products may be used for flea control when the mated bitches are placed in newly packed nest boxes in mid-April; at the end of June/early July when the kittens are moved to newly packed nest boxes and in the autumn when the animals for breeding are selected and gathered). In early March when the farmer is preparing for the mating the phoxim treatment is the most preferred. It should be noted that the latter two flea control products should not be used at the same animal or nest box and that there is a need for the treatment chosen to be repeated after 1 month.

## Conclusions

The treatment of a 0.1% aqueous phoxim dilution sprayed on the animals or with 4 grams of the 1.25% bendiocarb dusted in the next box material was highly efficient in reducing the number of fleas applied to the nesting materials experimentally before treatment. It is suggested that these compounds are implemented in a yearly flea management plan on mink farms.
